# Pathogenesis, Molecular Genetics, and Genomics of *Mycobacterium avium* subsp. *paratuberculosis*, the Etiologic Agent of Johne’s Disease

**DOI:** 10.3389/fvets.2017.00187

**Published:** 2017-11-06

**Authors:** Govardhan Rathnaiah, Denise K. Zinniel, John P. Bannantine, Judith R. Stabel, Yrjö T. Gröhn, Michael T. Collins, Raúl G. Barletta

**Affiliations:** ^1^School of Veterinary Medicine and Biomedical Sciences, University of Nebraska, Lincoln, NE, United States; ^2^Infectious Bacterial Diseases, National Animal Disease Center, USDA-ARS, Ames, IA, United States; ^3^Population Medicine and Diagnostic Sciences, College of Veterinary Medicine, Cornell University, Ithaca, NY, United States; ^4^Pathobiological Sciences, School of Veterinary Medicine, University of Wisconsin-Madison, Madison, WI, United States

**Keywords:** *Mycobacterium avium* subsp. *paratuberculosis*, Johne’s disease, pathogenesis, transposon mutagenesis, mutant bank

## Abstract

*Mycobacterium avium* subsp. *paratuberculosis* (MAP) is the etiologic agent of Johne’s disease in ruminants causing chronic diarrhea, malnutrition, and muscular wasting. Neonates and young animals are infected primarily by the fecal–oral route. MAP attaches to, translocates *via* the intestinal mucosa, and is phagocytosed by macrophages. The ensuing host cellular immune response leads to granulomatous enteritis characterized by a thick and corrugated intestinal wall. We review various tissue culture systems, ileal loops, and mice, goats, and cattle used to study MAP pathogenesis. MAP can be detected in clinical samples by microscopy, culturing, PCR, and an enzyme-linked immunosorbent assay. There are commercial vaccines that reduce clinical disease and shedding, unfortunately, their efficacies are limited and may not engender long-term protective immunity. Moreover, the potential linkage with Crohn’s disease and other human diseases makes MAP a concern as a zoonotic pathogen. Potential therapies with anti-mycobacterial agents are also discussed. The completion of the MAP K-10 genome sequence has greatly improved our understanding of MAP pathogenesis. The analysis of this sequence has identified a wide range of gene functions involved in virulence, lipid metabolism, transcriptional regulation, and main metabolic pathways. We also review the transposons utilized to generate random transposon mutant libraries and the recent advances in the post-genomic era. This includes the generation and characterization of allelic exchange mutants, transcriptomic analysis, transposon mutant banks analysis, new efforts to generate comprehensive mutant libraries, and the application of transposon site hybridization mutagenesis and transposon sequencing for global analysis of the MAP genome. Further analysis of candidate vaccine strains development is also provided with critical discussions on their benefits and shortcomings, and strategies to develop a highly efficacious live-attenuated vaccine capable of differentiating infected from vaccinated animals.

## Introduction

*Mycobacterium avium* subsp. *paratuberculosis* (MAP) is the etiologic agent of Johne’s disease (JD) in ruminants, a chronic enteritis with significant economic impact and worldwide distribution (1). The potential linkage of MAP to Crohn’s disease (CD) in humans continues to be intensively investigated with dissimilar results (2–4). MAP was first isolated by the German scientists Johne and Frothingham in 1895 (5). It causes disease primarily in ruminants (cattle, sheep, goats, deer, etc.), but there are also reports of infection in non-ruminants, especially in wildlife (6). In the United States, annual losses to the cattle industry have been estimated from $250 million (7) to $1.5 billion (8). A recent analysis of published data using a Bayesian method (9), adjusting for sensitivity and specificity, determined that the true United States dairy herd-level prevalence of MAP was actually 91.1% compared to the 70.4% reported in 2007 (10). In beef cattle, herd-level prevalence of MAP is 7.9% (11). Even though JD was first observed in the United States in the early 1900s, the focus on research and disease control has only increased in the past 20 years. A Voluntary Bovine JD Control Program is in place to control JD on farms and identify herds with a low risk of infection. Currently, one of the most cost-effective and sensitive testing methods for JD is the identification of MAP in herds by testing environmental fecal samples by culturing from high traffic areas (9). Indeed, the use of improved diagnostics coupled with good management practices have shown to decrease JD transmission (12). Unfortunately, wildlife reservoirs may undermine efforts to control JD in domesticated animals until their role in wildlife is fully understood (13).

## Taxonomy and Properties

**Figure 1:**
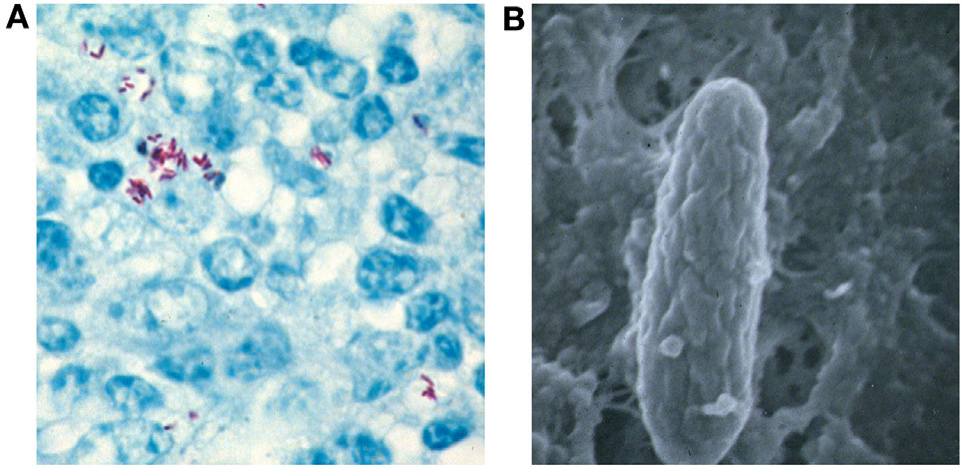
*Mycobacterium avium* subsp. *paratuberculosis* (MAP) properties. **(A)** Acid-fast stain of intestinal epithelium from an experimentally challenged bovine reveals MAP (red rods) inside macrophages. **(B)** Electron microscopy clearly shows the rod-shaped mycobacteria magnified over 50,000 times.

Mycobacterium avium subsp. paratuberculosis is part of the *Mycobacterium avium* complex in the genus *Mycobacterium* and family *Mycobacteriaceae*. The *M. avium* complex contains two clearly defined species *M. avium* and *M intracellulare*. *M. avium* is classified into four subspecies based on the comprehensive sequence-based analysis of the 16S-23S ribosomal RNA internal transcribed spacer (14, 15): *M. avium* subsp. *avium, M. avium* subsp. *hominissuis* (MAH), MAP, and *M. avium* subsp. *silvaticum*. MAP is a facultative intracellular, Gram-positive, acid-fast (Figure 1A) and small (0.5 × 1.5 μm) rod-shaped (Figure 1B) bacterium. The cell wall is thick and waxy and made up of mycolate and peptidoglycan layers held together by arabinogalactan. It is a slow-growing bacteria with a generation time of over 20 h (16). Initial attempts to cultivate MAP in laboratory media were unsuccessful (17) and it was hypothesized that the inability of MAP to grow under *in vitro* conditions may be due to the lack of some essential growth factor. Later, it was shown that MAP was able to grow in media supplemented with extracts from other mycobacteria (18, 19) and they concluded that MAP lacked the ability to synthesize some essential growth factor that is synthesized by other species. Mycobactin, an iron-binding siderophore isolated from *Mycobacterium phlei*, was shown to be the growth factor that is essential for the *in vitro* cultivation of MAP (20, 21). Since that time, mycobactin dependency has been considered to be taxonomic for MAP. More recently, with the genome sequence discussed further below, a molecular understanding of mycobactin dependency has been discovered by a deletion in the *mbtA* gene within the mycobactin synthesis operon (22, 23).

## Pathogenesis

**Figure 2:**
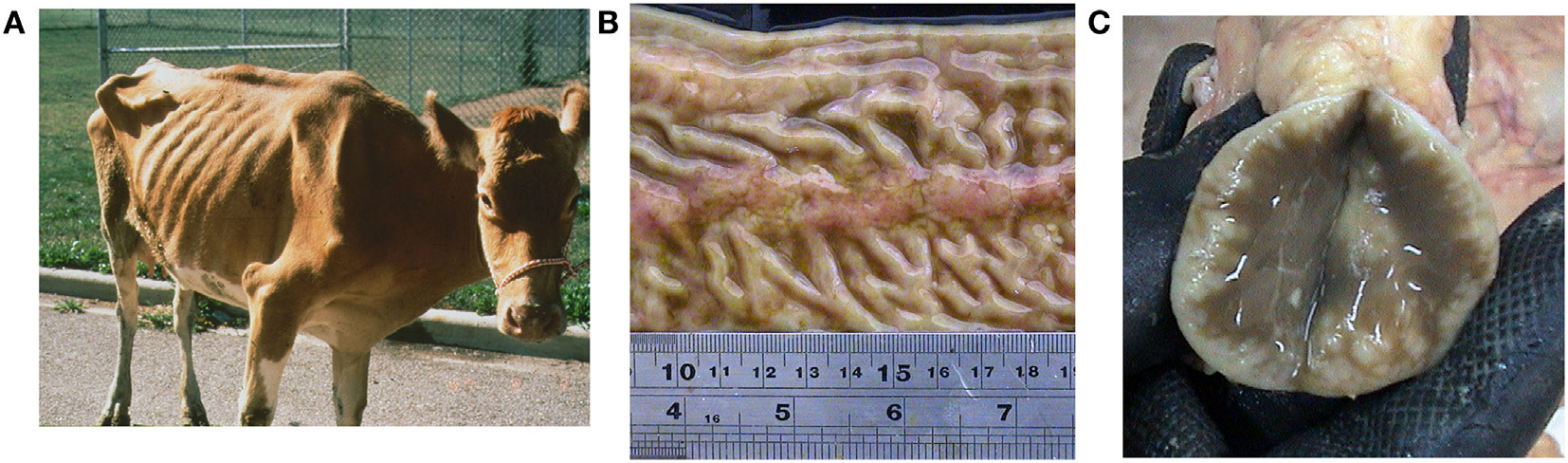
Johne’s disease affected animal caused by *Mycobacterium avium* subsp. *paratuberculosis*. **(A)** Severely debilitated cow with common symptoms of chronic diarrhea, malabsorption, muscular wasting, and malnutrition. The host cellular immune response leads to the typical granulomatous enteritis seen as thickening of the **(B)** intestinal mucosa with prominent Peyer’s patches, and **(C)** lymph node showing hyperactive lymphoid tissue (white spots).

Johne’s disease causes a chronic diarrhea characterized by a malabsorption syndrome that leads to malnutrition and muscular wasting (Figure 2A). Neonates and juvenile animals are infected mainly *via* the fecal–oral route. Transmission may also occur by the consumption of milk and colostrum from infected cows (24). Calves up to 6 months of age are at higher risk of getting infected, but the risk of infection drops after this age (25). Mouse models have shown that, after ingestion, MAP attachment to and translocation through the intestinal mucosa is mediated by both M-cells and enterocytes (26). Moreover, studies in tissue cultures demonstrated that MAP affects the formation of tight junctions in the intestinal mucosa providing a mechanism for increased permeability (27). There is significant host–pathogen crosstalk during this process as antigens 85 (28), 35 kDa (29), MAP oxidoreductase (30), MAP fibronectin-binding protein (31, 32), and the histone HupB (33) play important roles in MAP epithelial cell adhesion and/or invasion. Previous findings using a co-culture of the bovine mammary epithelial cell line MAC-T (34) and bovine blood-monocyte-derived macrophages (BMDM) suggest that phagosome acidification in MAP-infected epithelial cells leads to interleukin (IL)-1β production, macrophage recruitment, and transepithelial migration (35). Bacilli are subsequently phagocytosed by these sub- and intra-epithelial macrophages (36–38). Once inside phagocytic cells, the ability of MAP to survive and replicate within these phagocytic cells plays a key role in pathogenesis (39, 40). Moreover, use of a culture passage model showed that MAP lipid composition changes in macrophages developing a pro-inflammatory phenotype (41).

The ensuing host cellular immune response leads to the typical granulomatous enteritis pathognomonic of JD (38), characterized by the thick and corrugated appearance of the intestinal wall (Figure 2B) and inflamed lymph nodes (Figure 2C). Tissue macrophages and dendritic cells play a crucial role in the recognition of pathogen-associated molecular patterns in the innate phase *via* toll-like receptors (42), as well as in antigen processing and the elicitation of cytokine-mediated cellular interactions (43). Control of MAP infections are dependent on a T helper cell, Th1, type response and the subsequent activation of macrophages by interferon-gamma (INF-γ) secreted by Th1 T lymphocytes in the acquired immunity phase (44, 45). The killing mechanism of these activated phagocytic cells involves the generation of nitric oxide by the inducible nitric oxide synthase, whose activity has been already demonstrated in cattle (46). In this context, the overproduction of nitric oxide is particularly high in BMDM isolated from subclinically infected animals (47). However, MAP also affects the function of bovine macrophages as evidenced by unique mRNA expression profiles (48), inhibition of apoptosis and antigen presentation (49), and patterns of cytokine expression that have diagnostic significance (50). Predominantly, MAP drives T helper cells from infected cattle to undergo a Th2 response with enhanced expression of IL-4, IL-5, IL-10, and inhibitors of tissue remodeling factors (51, 52). This humoral response was confirmed in a neonatal calf model (53). Other findings also implicate regulatory T and Th17 cells in the immunopathogenesis of JD in both ruminants and wildlife (49, 54).

Several models have been developed to study MAP pathogenesis. However, MAP elicits responses from the ruminant host immune system that are not manifested in traditional *in vitro* models. For example, in infected BMDM, MAP bacilli multiply over 4–8 days (44, 55), but infection of the murine J774 macrophage cell line results in a decrease in bacterial loads over time (56, 57). Thus, primary phagocytic cells are preferred to conduct experiments when studying the interaction of MAP with phagocytic cells. A promising systems biology approach based on ileal loops has been developed to follow MAP infection from early to late stages (58). Recently, this model has been applied to compare the host transcriptome profile upon *M. avium* subsp. *avium* and MAP infection. Analysis of cellular pathways affected during MAP infection was consistent with intestinal mucosal weakening, the activation of a Th2 response, and phagocytosis inhibition that was not observed with *M. avium* subsp. *avium* (59).

## Diagnosis and Control

Infected animals shed MAP in the feces before showing any clinical signs, thus acting as a major source of infection to other animals in the herd. Early diagnosis of the infection is very important to prevent the spread of JD. Various diagnostic tests were developed based on the direct and indirect detection of MAP (60). The direct detection of MAP in clinical samples can be accomplished by (i) microscopy, (ii) MAP isolation by culturing, and (iii) the identification of MAP DNA by PCR. Ziehl–Neelsen or acid-fast staining has been used for the examination of clinical samples (Figure 1A). Acid-fast staining is the simplest, fastest, and most economical method of diagnosis, but the specificity and sensitivity are low as it is difficult to differentiate between MAP and other acid-fast bacilli (61). Ziehl–Neelsen staining can be used for the initial screening of MAP, but it has to be confirmed by other specific tests, such as PCR and/or immunoassays. The isolation of MAP by culturing is the “gold standard” for JD diagnosis. The dependence of MAP on mycobactin J for growth in specialized laboratory media can also be used to discriminate MAP from other acid-fast bacilli. Recently, an improved growth medium was described that improves MAP recoverability and sensitivity by 1,000-fold (62). Culture-based diagnosis is very time consuming as MAP grows extremely slow (6–8 weeks for colony formation on solid media). Therefore, a very rapid and sensitive PCR-based testing was used for MAP detection in clinical and environmental samples (63–65). This PCR utilizes primers for a 1.4 kb multicopy insertion element, IS*900*, which is sequence specific to MAP (60, 66). However, the presence of IS*900*-like insertion sequences in other mycobacteria was shown to compromise the specificity of this assay leading to false-positive results (64, 67). To overcome false-positive results, multiplex PCR based on the IS*900*, IS*901*, IS*1245*, and *dnaJ* gene has been developed, but the sensitivity of this test is low due to reagent interference and primer dimer formation (60, 68). In addition, PCR assays with fecal samples are only 70% sensitive and 85% specific (69). Some good progress has been made identifying and using more specific targets for PCR tests (70–72), and this comparative genomic approach has filled a knowledge gap in MAP diagnostics. Some of these targets have even been used in commercial diagnostic kits.

Diagnostic MAP assays created on the indirect detection are based on the host immune response to infection. The delayed-type hypersensitivity skin test has been developed using a Johnin purified protein derivative (73). However, this test is not specific as other environmental mycobacteria can also sensitize the animal and give false-positive results. So delayed-type hypersensitivity skin tests cannot distinguish vaccinated from naturally infected animals. As stated previously, MAP infection leads to a T helper cell response which secrets INF-γ. The measurement of this INF-γ level by an enzyme-linked immunosorbent assay (ELISA) can also be used for the diagnosis of JD using day-old blood sample culture supernatants stimulated with Johnin and co-stimulated with bovine IL-12 and/or human IL-2 (74). Unfortunately, MAP purified protein derivatives are also used as antigens in the INF-γ assay leading to problems with cross-reactivity. The cell wall lipopeptide, L5P, has been studied as an alternative MAP antigen for the assay, but the INF-γ response has been shown to be lower than with Johnin (75). Thus, routinely used methods for MAP diagnosis include the detection of antibodies in serum and milk from infected animals using a commercial ELISA kit: (i) HerdCheck *M. paratuberculosis* ELISA (IDEXX Laboratories, Inc.), (ii) ParaCheck (CSL/Biocor), (iii) SERELISA ParaTB (Synbiotic Corp.), and (iv) ID Screen^®^ Paratuberculosis Indirect (IDvet Genetics). Compared to PCR diagnosis, an ELISA has a lower sensitivity of 50%, but a much greater specificity of 99.8% (76, 77). More studies are required to identify MAP specific antigens to develop immune-based assays for diagnosis of JD that have a higher level of sensitivity.

Control measures to prevent JD include vaccination (the most cost-effective), testing, and improved herd management based on a producer’s resources, facilities, and operation (78). Unfortunately, though there are JD vaccines that reduce clinical disease and shedding, their efficacies are limited and none afford long-term protective immunity. For example, in the United States, Mycopar^®^ (Boehringer Ingelheim Vetmedica, Inc.) is the only licensed vaccine against JD in cattle. However, this vaccine was derived from *M. avium* subsp. *avium* strain 18 (79) and; therefore, does not have an optimal antigenic repertoire. Another bacterin, Silirum^®^ (Zoetis Animal Health) is being tested in Australia and approved for limited use in cattle. This vaccine contains the heat-killed MAP 316F strain. This formulation may possess a better antigenic repertoire, but while using heat-killed bacteria may improve safety, it may also reduce efficacy. Neoparasec^®^ (Rhone-Merieux) contains the live-attenuated MAP strain 316F while Gudair^®^ (Zoetis Animal Health) is heat-killed 316F and both are licensed for use in sheep and goats. However, current vaccines cannot distinguish vaccinated from infected animals, thus compromising JD diagnostic tests (80), and strain 316F was generated in the 1920s by random attenuation procedures (e.g., passages on ox bile) that are only now being investigated (81). In the final analysis, a vaccine of high efficacy is needed to significantly control JD (82).

Testing results on the new generation of human anti-tuberculosis vaccines seem to indicate that live-attenuated vaccines provide better protection than subunit vaccines (83). As JD is caused by a *Mycobacterium*, it is likely that a similar situation will occur for candidate subunit or bacterin-based vaccines. This was actually the impetus behind the JD Integrated Program—Animal and Plant Health Inspection Service efforts to undertake a standardized vaccine test program. United States and New Zealand researchers contributed 22 blinded live-attenuated vaccine candidates to be evaluated in a three-phase study: BMDM, mouse, and goat models. Though the methodology for animal testing was well developed (84), most of the attenuated transposon mutants tested were first-generation ones and carried the Tn*5367* transposase which lead to instability. Moreover, there were unknowns that could not be ascertained before the start of the trial, such as the best immunization route and dosing schedule. Nonetheless, important data and reagents were developed (80). It may still be possible to develop a subunit vaccine capable of controlling infections by eliciting the appropriate type of humoral immunity (85), especially against antigens expressed in the pro-inflammatory phenotype (86).

## Zoonotic Potential

Mycobacterium avium subsp. paratuberculosis is among a list of pathogens that have been associated withCD, a human chronic inflammatory bowel disease (2, 87, 88). The cross-reactivity of MAP antigens with those in humans, such as the zinc transporter 8 protein, may underlie the etiology of these diseases (89). MAP is also postulated to be involved in the progression of HIV infection and other immune dysfunction diseases: multiple sclerosis, sarcoidosis, type 1 and 2 diabetes mellitus, Hashimoto’s thyroiditis, and Parkinson’s disease (90–96). Over the years, several studies have investigated the role of MAP in CD with conflicting results (4, 97–100). MAP has been isolated from CD patients by culturing blood, breast milk, and tissue biopsy samples, and samples were shown positive for IS*900* by PCR (101–103). Also by this method, MAP was present in the gut of 92% of CD patients and 26% of control individuals (98). Humans are exposed to MAP from various sources that could be contaminated from infected animals, such as drinking water with feces, milk, etc. (104). In the United Kingdom, a study revealed that even after pasteurization, MAP was detected by IS*900* PCR in 7% of retail milk for human consumption (105). Nonetheless, this is highly dependent on the pasteurization method since the application of a standard high-temperature short-time pasteurization results in the destruction of MAP (106). However, some bacteria survived the sub-pasteurization heat treatment at lower temperatures used for cheese manufacture. Thus, though humans are widely exposed to MAP, susceptible individuals with a compromised immune system, due to other illness or genetic factors, may be at higher risk for infection and disease. In these individuals, MAP may survive and multiply inside gut macrophages leading to immune dysregulation and inflammation of the intestinal wall resulting in a “leaky gut” (http://www.crohnsmapvaccine.com). Microbes and food materials penetrate the leaky gut causing massive wall inflammation leading to chronic inflammatory bowel disease.

Currently a combination of anti-inflammatory, anti-mycobacterial, and immunosuppressant drugs are used or in trials for CD treatment. Initially, only anti-tuberculosis drugs were used for treatment of MAP infections in humans, but the results were not promising. Later macrolides (e.g., azithromycin and erythromycin), rifabutin, and clofazimine have been used with limited success, but the treatment regimen is very lengthy (2, 66, 107–109). A comprehensive study evaluated culture methods for the determination of MAP *in vitro* drug susceptibility and concluded that the BACTEC™ MGIT™ system was more rapid and reliable (110). In addition, *in vitro* studies using drug combinations of anti-microbials and immunomodulators displayed synergistic effects that could be applied to CD treatment (111). Therefore, more studies are needed to identify novel drugs and/or targets for the development of better therapeutics against MAP to shorten and simplify the treatment. Regarding MAP pathogenesis, the European Food Safety Authority has conducted a thorough study and indicated that MAP is eligible to be listed for Union intervention based on Article 5(3) of the Animal Health Law (112). Several species of mammals and birds were listed as susceptible species, including Bovidae, Cervidae, and Leporidae, but there was no consensus on the zoonotic risk of MAP in the context of human diseases, such as CD.

## Genomics

The MAP bovine strain K-10 genome has been sequenced, annotated, and re-examined by optical mapping (22, 113, 114). The updated K-10 genome is 4,829,781 bp encoding 4,350 protein-encoding ORFs with a 69.3% GC content. In the genome, about 60% of the ORFs have similar sequences in microbial genetic databases, but only approximately 35% have well predicted or identified functions (115). Nonetheless, about 75% of the MAP genes have counterparts in *Mycobacterium tuberculosis* with 39 predicted proteins that are unique to MAP. This genome possesses a high redundancy rate due to gene duplications, particularly for those involved in lipid metabolism and redox processes. Gene functions include 150 transcriptional regulators, 16 polyketide synthesis, 14 two-component regulatory systems, 16 serine–threonine protein kinases, 8 mammalian cell entry (mce), 20 insertion elements, and 5 putative prophages. Sequencing also identified and mapped genes involved in glycolysis, pentose phosphate pathway, tricarboxylic acid cycle, and glyoxylate cycle (116). Important differences between the genomes of K-10 and *M. tuberculosis* human strain H37Rv include the following: (i) no intact Pro-Glu-polymorphic GC-rich sequences (PE-PGRS) genes, (ii) 47 fewer Pro-Pro-Glu (PPE) genes in *M. tuberculosis* (117), and (iii) the presence of a truncated salicyl-AMP ligase gene (mtbA) which may explain the MAP mycobactin biosynthesis defect discussed earlier (22).

Other MAP strains with complete sequences include the human strain MAP4 (118) and another bovine strain JII-1961 (119). Partial MAP genomic sequences include 10-4404, 2015WD-1, 2015WD-2, ATCC19698, JQ5, S5, S397, JIII-386, and several human isolates (118, 120–122). The MAP ovine genomes of strains S397 and JIII-386 differ by one inversion to the MAH human strain 104, while two inversions are detected between K-10 and MAH, with additional differences involving duplications, deletions, and polymorphisms. Based on these analyses, ovine isolates seem to be an intermediate in the evolution of MAP bovine strains from MAH human strains (115), although this is not definitely established (121). However, the more closely related bovine and human isolates (118, 120) may be the latest strains that diverged in evolution acquiring specific host adaptations in their genomes.

## Transposon Mutagenesis

**Figure 3:**
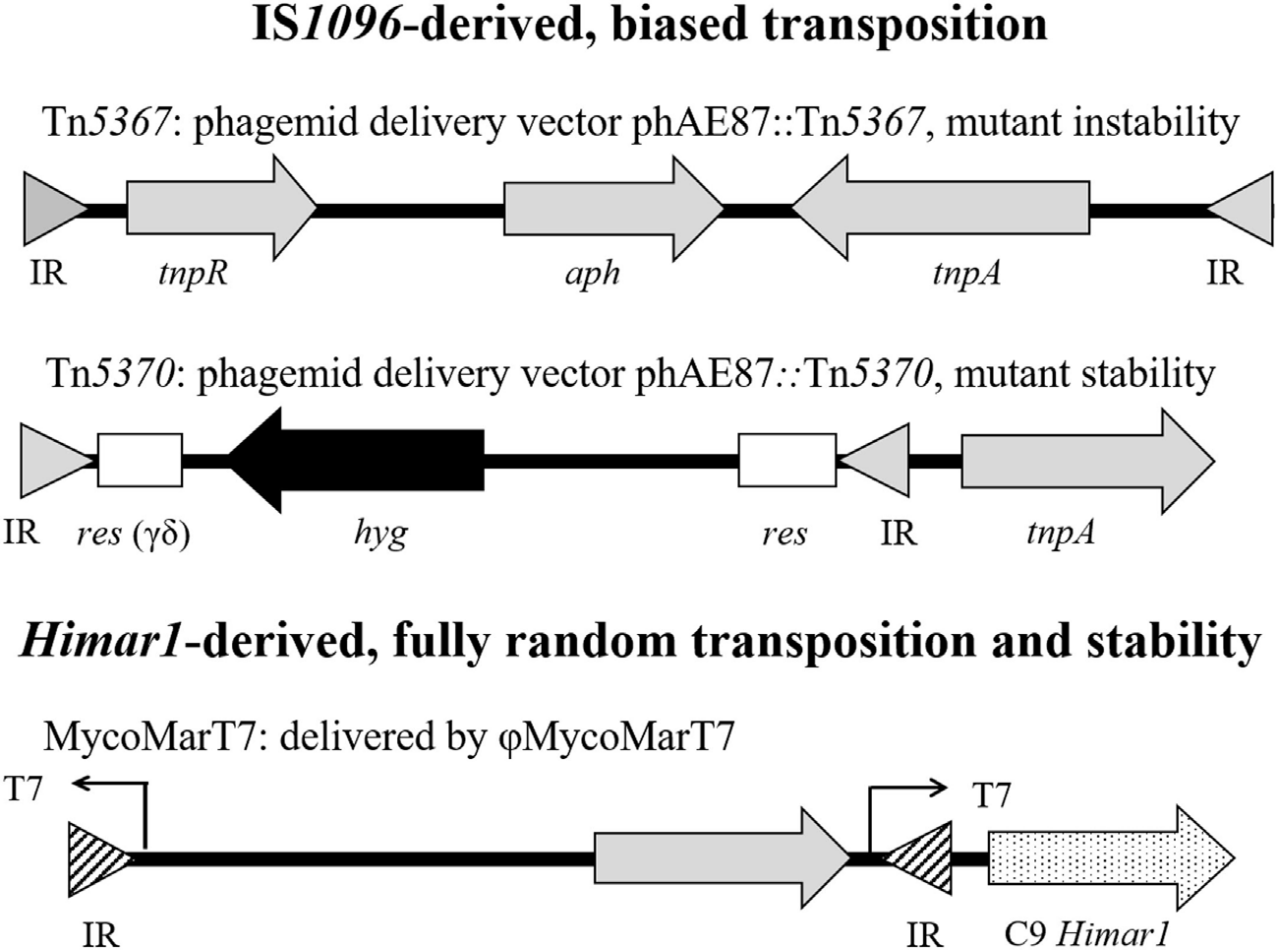
Structure of mycobacterial transposons utilized to generate random mutant libraries. Inverted repeat (IR) for Tn*5367* or Tn*5370* (filled triangle), or *Himar1*-derived transposon (striped triangle); *tnpR*, IS*1096* resolvase; *aph*, kanamycin-resistant gene; *tnpA*, IS*1096* transposase; *res*, resolution site for transposon gamma-delta; *hyg*, hygromycin resistant gene; and C9 *Himar1, Himar1* transposase.

The identification and functional analysis of genes involved in the pathogenesis of MAP requires molecular genetic tools to manipulate its genome. Because the MAP cell wall is very thick and impermeable, it is difficult to introduce plasmid vectors. The shuttle plasmid vector pMV262 carrying a mycobacterial origin of replication (*oriM*) and an *Escherichia coli* origin of replication (*oriE*) have been used to transform MAP (123). However, the transformation efficiency was very low; therefore, a phage-mediated transduction process was developed to introduce DNA into MAP (124). Shuttle phasmids (phagemids) carrying the mycobacteriophage TM4 (125) and an *E. coli* cosmid have been widely used for the genetic manipulation of MAP. More recently, the generation of MAP mutant libraries using transposon mutagenesis has been the major approach to define virulence determinants and identify genes that are essential and non-essential (126). Two types of transposons have been extensively used in mycobacteria to generate random mutant banks: (i) IS*1096*-derived and (ii) *Himar1*-derived transposons (Figure 3).

Tn*5367* (GenBank Accession KM232614) was the first transposon widely used in mycobacteria including MAP (126–128). This transposon (3,381 bp) carries a kanamycin-resistant drug marker (*aph*), and the IS*1096* transposase (*tnpA*) and resolvase (*tnpR*). This transposon has the transposase within the transposed element (126) resulting in a relatively high transposition frequency (ca. 1.0 × 10^−5^), at least in *Mycobacterium smegmatis* (129). Tn*5367* transposes by a cutting and paste mechanism (130) and may insert in another gene creating either a wild-type or mutated gene, depending on precise or imprecise excisions, in the location of the original insertion. The transposon can be delivered into *Mycobacterium* by the thermosensitive phasmid derivative of mycobacteriophage TM4 (126). Since the IS*1096* transposase is located within the transposed element, the corresponding MAP mutants may not be stable enough to conduct long-term *in vivo* experiments where bacilli multiply to large numbers. An improved IS*1096*-derived transposon, Tn*5370* (GenBank Accession KM232615), was constructed by removing ORFs dispensable for transposition (e.g., *tnpR*), therefore, reducing the size to 2,295 bp (128, 130). Moreover, the transposase was engineered into the phasmid outside of the transposable element. Thus, Tn*5370*, as opposed to Tn*5367*, generates stable mutants upon transposition. This transposon has the advantage of possessing a hygromycin (*hyg*) marker within the transposable element outflanked by the γ-δ resolvase. This construction allows for the excision of the drug marker and the generation of unmarked transposon insertion mutants. However, neither Tn*5367* nor Tn*5370* transposes randomly in mycobacteria (127, 130).

*Himar1*-derived transposons have also been used to generate random mutant libraries in *M. tuberculosis* and MAP (131–134). The *Himar1* transposon (MycoMarT7) was engineered into the same TM4 phasmid derivative described above. This vector carries the highly active C9 *Himar1* transposase outside the inverted repeats, thus yielding stable mutants. In addition, this vector carries the *aph* drug marker and a T7 promoter that reads outward to facilitate identification of insertion sites by *in vitro* transcription and cDNA synthesis (131). The major advantage of the *Himar1* derivative is the almost fully random recognition sequence (5′-TA-3′) versus the recognition sequence of IS*1096*-derived transposons (5′-NNPy(A/T)A(A/T)NN-3′) showed experimentally to have a transposition bias in both *M. tuberculosis* and MAP (127, 130). Most of the MAP transposon mutant libraries were made with Tn*5367* (127, 128, 135, 136) and fewer libraries have been constructed using the *Himar1*-derived transposon MycoMarT7 (132, 133). Our group has constructed a *Himar1* library for MAP and used it to demonstrate random genomic insertions (134).

## Molecular Genetics in the Post-Genomics Era: Candidate Vaccine Applications

Our understanding of MAP pathogenesis has greatly improved based on the completion of the MAP K-10 genome sequence, and the availability of advanced molecular biology and bioinformatics tools. MAP genomics has many applications, such as molecular detection, determining the molecular evolution of MAP as a pathogen, and identifying virulence determinants, drug targets, attenuation targets for vaccine development, and/or diagnostic antigens. Several strategies were used to mine the MAP genome for virulence determinants or important diagnostic antigens (137–139). One approach relied on identifying MAP genes with known *M*. *tuberculosis* homologs known to play important roles in pathogenesis (140, 141). Many of these studies described below have generated deletion mutants to identify the role of these genes in pathogenesis. Usually, phasmids are used to deliver the allelic exchange substrate into MAP to produce the desired gene deletion by homologous recombination. For example, based on the attenuation of *M*. *tuberculosis leuD* mutants (140), the corresponding MAP *leuD* mutants were constructed by allelic exchange, characterized by carbon and nitrogen source utilization, and showed to be attenuated in mice (142, 143). Likewise, a mutant of MAP *lipN* was constructed and displayed lower levels of colonization in mice compared to the wild-type strain (144). Similarly, *lsr2, pknG*, and *relA* deletion mutants were generated (145) and later *in vivo* mice studies revealed that *relA* mutants were immunogenic, but had decreased pathogenicity (146). Other studies constructed *sigH* and *sigL* deletion mutants, infecting bovine macrophages and mice to demonstrate that this gene plays a role in pathogenesis (147, 148). Recently, *sigH* and *lipN* deletion mutants were validated as promising live-attenuated vaccine candidates in goats (149). Nonetheless, these meritorious studies are not comprehensive since genetic islands were identified in the MAP genome demonstrating a gene organization different from the closely related MAH (150). Indeed, differences in gene organization may lead to a context-dependent function (e.g., even homologous genes in MAP and MAH may play different roles in pathogenesis). This effect is further compounded by findings indicating host genome adaptations (115).

In the context of vectored subunit vaccines, success has been achieved in the expression of MAP antigens in *Lactobacillus salivarius* using a codon optimization strategy (151, 152). Multi-antigen virally vectored vaccines were also engineered and tested in mice and cattle. In this context, the vaccine designated HAV is a fusion of two secreted and two cell surface MAP proteins whose DNA coding sequence was inserted into the human adenovirus 5 and Modified Vaccinia Ankara delivery vectors. Vaccination of naive C57BL/6 mice resulted in a highly immunogenic response with significant levels of IFN-γ and a humoral response against the recombinant antigens (153). The vaccine provided moderate protection against challenge. This vaccine was also tested in cattle with promising results being well tolerated, showing no cross-reactivity against bovine tuberculin, providing some protection against challenge in a calf model and eliciting CD4+, CD8+ IFN-γ producing T-cell populations and, upon challenge, developed early specific Th17 T-cells (154). In addition, a non-recombinant *Lacotbacillus casei* used as a probiotic immunomodulated and reduced pathology in a mouse MAP infection model (155). Moderate success has been attained with recombinant MAP Hsp70 proteins resulting in a reduction of bacterial shedding upon experimental infection without compromising the diagnosis of bovine tuberculosis (156, 157) and showed some promise as a therapeutic vaccine in cattle (158). Lipoarabinomannan-enriched glycolipid extracts used as potential subunit vaccines against MAP yielded a pro-apoptotic response in bovine macrophages and reduced shedding (159, 160). Studies have also been conducted with purified antigens or antigen cocktails. For example, superoxide dismutase and antigen 85B were shown to elicit T-cell responses consistent with protection in mice (161, 162). These results were further advanced using DNA vector vaccines (145). Various MAP protein cocktails were also tested in mice and cattle, showing reduced tissue burden (163), and INF-γ and humoral responses, respectively (164). Nonetheless, the extensive experience of researchers in the human tuberculosis vaccine field has led to the consensus that live-attenuated vaccines are the best approach for vaccination against mycobacterial diseases (83).

Gene expression in MAP is complex and global regulation has been associated with 19 putative sigma factors with 12 belonging to common mycobacterial gene families. Specific sigma factors may be involved in the expression of survival and virulence genes (165). In addition, MAP virulence determinants are regulated by the LuxR–LuxI quorum sensing system (166). Transcriptomic analysis has been applied extensively and these studies generated large expression databases under conditions of environmental stress (144), iron limitation (167), intracellular survival within BMDM, tissue localization *in vivo* (168), and ligated jejuno-ileal loops (58, 59). Some differentially regulated genes such as *atpC, clpB, dnaJ, dnaK, groEL2, infB, kdpE, mbtC*, and *pmmA* correspond to essential genes in *M*. *tuberculosis* (131, 144, 169). Other genes identified as non-essential more likely to be involved in pathogenesis rather than general physiological functions: *aceAB, clpX, relA, htpX, lipL, lipN*, and *lpqP*. Indeed *M*. *tuberculosis* homologs of *lipL, lipN, aceAB*, and *lpqP* were also identified as being required for intracellular survival within macrophages by a genetic approach (170). Another insight from transcriptomic studies revealed significant differences in gene expression with MAP metabolic genes being shut down in tissues, while phagocytic cells upregulated genes involved in iron acquisition and intracellular survival (168). Interestingly, the analysis of databases from different studies does not reveal any significant correlations. Moreover, the fold-changes in gene expression levels differed widely from one study to another. Nonetheless, except for specific and restricted conditions, a common feature is that approximately 20–25% of the genes in the MAP genome change their expression levels based on the condition. Likewise for *M*. *tuberculosis*, little correlation was observed among genes that were up- or downregulated in macrophages or infected mice, and those identified by a genetic approach as being required for survival in macrophages and mice (170, 171). In retrospect, these results are not surprising as genetic methods establish gene requirements for a certain condition as an outcome of a cumulative selection process during the infection, independent of their mode of regulation. In this context, transcriptomic approaches may miss a significant number of constitutively or transiently expressed genes involved in pathogenesis. As transcriptomic approaches require discrete rather than continuous samplings, most appropriate time points could be missed.

Transposon mutagenesis is another strategy that has been used to identify MAP virulence genes. In this approach, genes are disrupted using random transposition to generate a mutant library where each mutant is marked with a transposon. Mutants are then screened under different *in vitro* and *in vivo* conditions for the functional analysis of genes. Mutants that have a mutation in genes that are essential will not survive. A library of 5,060 Tn*5367* transposon mutants was generated to apply a transposon–chromosome junction site sequencing protocol in order to identify the disrupted genes (127). They analyzed 1,150 mutants and identified 970 unique insertion sites. Based on sequencing analysis, they selected 11 mutants for mouse infection experiments and identified some potential virulence genes (*gcpE, pstA, kdpC, papA2, impA, umaA1*, and *fabG2_2*). In our study, we generated and screened a MAP transposon library of 13,536 mutants, identifying genes potentially involved in pathogenesis (e.g., MAP1152, MAP1156, MAP1566, and *lsr2*) (128). Another study used the *Himar1* transposon in MAP to isolate 111 mutants attenuated in BMDM from a random screen of 2,290 (132). Unfortunately, all of these studies have limitations: (i) the screening and analysis methods used are laborious and time consuming, especially for slow-growing MAP and (ii) the library used for screening is partial because of transposon bias (134) so it is not possible to perform the functional analysis of the entire MAP genome. To overcome these limitations, it is necessary to generate a comprehensive MAP mutant library and design new and more robust approaches to perform global gene functional analysis.

Transposon site hybridization (TraSH) has been applied for the global analysis of the *M. tuberculosis* genome (131). In this method, a high-density transposon mutant library is generated and the mutants are pooled. DNA microarray is then applied to map all transposon insertion sites. This approach has some limitations, such as poor resolution, limited dynamic range, and the requirement to develop DNA microarrays. Recently, a new method has been established based on next-generation deep sequencing to analyze the essentiality of genes on a whole-genome basis called transposon sequencing (Tn-Seq) (172). Tn-Seq involves the generation of a comprehensive mutant library using a transposon that inserts randomly without any biases. The mutants are pooled to isolate genomic DNA so the transposon chromosomal junctions can be amplified and sequenced using an Illumina or another suitable platform. This approach has been applied for *M. tuberculosis* to define essential genes under various conditions (173, 174). More recently, the Tn-Seq method was used to classify MAP essential genes by generating a pool of approximately 100,000 *Himar1* transposon mutants (133). However, we have demonstrated that *Himar1* transposons recombine with significant loci-dependent biases, making these collections highly underrepresented (134). The application of the Tn-Seq system to a highly saturated *Himar1* transposon mutant library will overcome these limitations and provide a global approach to the identification of MAP essential genes and virulence determinants.

## Perspective and Overall Summary

Sequencing the MAP K-10 genome, and the availability of advanced molecular biology and bioinformatics tools have allowed the screening and characterization of transposon mutants to identify specific genetic loci involved in pathogenesis, suggesting novel strategies for the control and diagnosis of JD. In addition, several candidate genes for strain attenuation have been mutagenized by allelic exchange and the corresponding mutants were tested with promising results in mice, goats, and cattle. We consider that vaccination with a live-attenuated vaccine is the most cost-effective control measure for MAP infection in livestock. The embodiment of such a vaccine would require the construction of a strain with two deletion mutations and with the capability to differentiate vaccinated from infected animals.

In summary, this article opens with a brief review of JD epidemiology and the staggering economic losses followed by MAP taxonomy and cultivation. The main mechanisms of pathogenesis related to the invasion of the intestinal epithelium and the underlying humoral and cellular responses are discussed. Infection models are concisely summarized thereafter. The article then shifts to the multiple methods of JD diagnosis, including acid-fast stain, culture, PCR, and the use of ELISA to detect the antibody and INF-γ responses. The control of JD is explained in the context of commercially available vaccines and their efficacies. This section also includes results of a recent multi-institutional live-attenuated JD vaccine testing program. MAP zoonotic potential is described based on the diagnostic methods used to detect MAP in human samples along with the various treatment options. Subsequently, we focus on genomics, including the analysis of bovine and ovine genome sequence isolates. This leads to the transposon mutagenesis section where we define the transposons and vectors used and summarize the results obtained with mutant banks. The final post-genomic section discusses the construction of new attenuated mutants by deletion mutagenesis and their vaccine potential. We also discuss antigen and antigen cocktails that have been suggested for subunit vaccine formulations. The manuscript concludes with the discussion of gene expression, global regulators, methods of wide genomic analysis, such as TraSH mutagenesis and Tn-Seq analysis, and our perspective on current and future vaccine efforts.

## Author Contributions

GR wrote excerpts of this review as Chapter 1 of his Doctoral Dissertation in Veterinary and Biomedical Science available online at the University of Nebraska-Lincoln (http://digitalcommons.unl.edu/). DZ, JB, JS, YG, MC, and RB contributed and edited the manuscript.

## Conflict of Interest Statement

The authors declare that the research was conducted in the absence of any commercial or financial relationships that could be construed as a potential conflict of interest.

## Abbreviations

MAP, *Mycobacterium avium* subsp. *paratuberculosis*; JD, Johne’s disease; MAH, *M. avium* subsp. *hominissuis*; BMDM, blood-monocyte-derived macrophages; IL, interleukin; INF-γ, interferon-gamma; ELISA, enzyme-linked immunosorbent assay; CD, Crohn’s disease; TraSH, transposon site hybridization mutagenesis; Tn-Seq, transposon sequencing; DIVA, differentiate vaccinated from infected animals.
